# Effects of lipocalin-2 on brain endothelial adhesion and permeability

**DOI:** 10.1371/journal.pone.0218965

**Published:** 2019-07-03

**Authors:** Yang Du, Wenlu Li, Li Lin, Eng H. Lo, Changhong Xing

**Affiliations:** 1 Department of Neurology, Xiangya Hospital, Central South University, Changsha, Hunan, China; 2 Neuroprotection Research Laboratory, Departments of Radiology and Neurology, Massachusetts General Hospital, Harvard Medical School, Charlestown, Massachusetts, United States of America; 3 School of Pharmaceutical Sciences, Wenzhou Medical University, Wenzhou, Zhejiang, China; 4 Department of Pathology, University of Texas Southwestern Medical Center, Dallas, Texas, United States of America; University of South Florida, UNITED STATES

## Abstract

Lipocalin-2 (LCN2) is a stress protein, and can be hyper-produced by many kinds of cells after exposure to injury or disease conditions. In this study, we asked whether LCN2 may play a protective role in cerebral endothelium. After focal cerebral ischemia in rats, plasma levels of LCN2 were significantly elevated at 6, 12, and 24 hrs, and persisted until 3 days post-stroke. To assess the vascular mechanisms of LCN2, we used brain endothelial cell cultures to investigate its effects on neutrophil adhesion and endothelial barrier integrity. LCN2 did not affect neutrophil adhesion to endothelial cells either under normal conditions or after TNFα stimulation. TNFα significantly increased endothelial permeability, and LCN2 rescued endothelial permeability. Concomitantly, LCN2 restored the membrane distribution of the tight junction protein ZO-1 and the adherens junction protein VE-cadherin. Our findings suggest that elevated LCN2 in the blood after ischemic stroke might affect endothelial function, in part by reducing damage to endothelial junctional proteins and maintain blood-brain barrier integrity.

## Introduction

Inflammation is a major component of stroke pathophysiology [1–3]. Although the underlying mechanisms are multi-cellular and multi-factorial, two cardinal features may always be present, i.e. perturbations in blood-brain barrier (BBB) integrity and the recruitment of peripheral immune cells into the injured central nervous system (CNS). For both of these phenomenon, cerebral endothelium should play a central role. Endothelial dysfunction results in a leaky BBB that worsens neuroinflammation [4]. Increased endothelial expression of adhesion molecules promotes the trafficking and infiltration of blood-borne cells including macrophages, neutrophils, and T lymphocytes [5]. Hence, finding ways to protect the cerebral endothelium is an important goal for stroke therapies.

Lipocalins are secreted proteins that play important signaling roles in many organ systems [6]. Many members of this superfamily comprise factors that are already known to contribute to CNS inflammation, including arachidonic acid and prostaglandins [7–9]. Therefore, it may not be surprising that lipocalin 2(LCN2) should also contribute to stroke pathophysiology. LCN2 was originally described as neutrophil gelatinase-associated lipocalin (NGAL) [10]. LCN2 or NGAL can bind and modulate the function of matrix metalloproteinases and their inhibitors, thus influencing neurovascular injury and remodeling [11–14]. More recently, it has been shown that LCN2 may also act directly in stroke. At high concentrations, LCN2 may promote neuronal cell death [15–17], but under some conditions, LCN2 can be released by injured neurons as a help-me signal that shifts microglia and astrocytes into neuroprotective phenotypes [18]. Besides, LCN2 may also function as an angiogenic factor [19, 20], thus potentially promoting neurovascular remodeling.

In the present study, we asked whether lipocalin 2 (LCN2) signaling may represent a potential target mechanism for ameliorating inflammatory changes in permeability and adhesion in the endothelium. First, we found that LCN2 levels in plasma significantly increased as early as 6 hours after cerebral ischemia in a rat middle cerebral artery occlusion model, and the elevation of LCN2 persisted till 3 days after cerebral ischemia. Then we asked whether the elevated LCN2 in the blood might play a role in promoting migration of peripheral immune cells into the ischemic brain via the changes of endothelial adhesion and integrity. Our data showed that (i) LCN2 did not affect the neutrophil adhesion to the endothelial cells either under normal condition or after TNFα treatment, (ii) LCN2 significantly reduced the endothelial permeability induced by TNFα treatment through increasing the membrane distribution of ZO-1 and VE-cadherin. Taken together, these results suggest that LCN2 may serve as an endogenous help-me signal to defend the cerebrovascular system after stroke.

## Material and methods

### Rat cerebral ischemia

All experiments were performed following a protocol approved by Massachusetts General Hospital Institutional Animal Care and Use Committee (IACUC) in accordance with the NIH Guide for the Care and Use of Laboratory Animals. Care is taken to minimize pain and discomfort throughout surgical procedures in the study. Deep anesthesia is induced prior to any procedure, and maintained throughout the surgery. All animals will be monitored daily for signs of infection, pain and distress. Analgesic medication will be provided for 3 days after survival procedures. Unhealthy animals will received prompt medical attention and treatment. Euthanasia or planned sacrifice will be achieved using 5% isoflurane inhalation. The euthanasia method follows approved IACUC protocols consistent with the recommendations of the Panel on Euthanasia of the American Veterinary Medical Association. All studies and measurements were randomized and blinded. Permanent focal cerebral ischemia was induced by monofilament occlusion of the middle cerebral artery in male Wistar (280-300g, Charles River Laboratories Wilmington, MA, USA) under 1.2% isoflurane anesthesia and with Laser Doppler monitoring. Rectal temperature was maintained at 37°C±1 with a thermostat-controlled heating pad. Blood pressure, pH and gases were all within normal range (blood pressure: 96.5±10.7 mmHg; pH: 7.42±0.05; pO2: 145±11 mmHg; pCO2: 37±2 mmHg). The blood was drawn at various time points after cerebral ischemia (3hr, 6hr, 12hr, 24hr, 3d, 7d, and 14d). and the plasma was obtained by centrifugation at 1500 rpm for 15 min. The levels of LCN2 in plasma samples were measured using ELISA kit (Abcam), according to manufacturer instructions.

### Human brain microvascular endothelial cell culture

Human brain microvascular endothelial cells (HBMECs) (Cell systems, Kirkland, WA) were cultured in flasks coated with rat tail collagen I (Corning, Bedford, MA) and maintained in endothelial basal medium (EBM)-2 (Lonza, Hopkinton, MA) supplemented with fetal bovine serum, fibroblast growth factor-2, epidermal endothelial growth factor, hydrocortisone, insulin-like growth factor, ascorbic acid, VEGF, and amphotericin B. Cells of passage 5 to 12 were used for the experiments. When 80–90% confluent, HBMECs were treated with 50ng/ml of recombinant human TNFα (R&D) with or without 1μg/ml of LCN2 (R&D, Minneapolis, MN) for 24hr. Cytotoxicity was measured by a standard lactate dehydrogenase (LDH) release assay (Roche, Germany), and proliferation was evaluated by standard 3-(4,5-dimethylthiazol-2-yl) 2,5-diphenyl-tetrazolium bromide (MTT) assay.

### Immunocytochemistry

The HBMECs cells were fixed with 4% paraformaldehyde for 30 min, and blocked with 5% normal horse serum for 1 hr. The cells were incubated with primary antibody against LCN2 receptors LRP2 (Abcam, Cambridge, MA) and 24p3R (Bioss, Woburn, MA) as well as ZO-1 (Thermo Fisher) and VE-cadherin (Cell signaling) at 4°C overnight. After washing, the sections or cells were incubated with Alexa Fluor 488- or 594-conjugated secondary antibodies (1:200, Molecular Probe, Grand Island, NY) for 1 hr at room temperature. Negative controls were incubated without primary antibodies and no immunoreactivity was observed in these controls.

### Neutrophil adhesion assay

The neutrophil adhesion assay was performed according to previously described methods [21]. SD rats were anesthetized and given an intraperitoneal (i.p.) injection of 5 ml of 3% thioglycolate media (Sigma, St. Louis, MO). Twenty four hours later, the rats were sacrificed and 150 ml of PBS was i.p. injected. After a massage of the abdomen, the peritoneal fluid was collected. MitroTracker Red CMXRos (Invitrogen Life Techologies) (1mM) was added to the peritoneal collection at a 1:1000 dilution and incubated for 1h in the incubator at 37°C, during which time macrophages adhere to the bottom of the dish and neutrophils remain floating in the media. The media containing the floating neutrophils were collected and centrifuged at 1000rpm for 5min. The pellets containing neutrophils were resuspended in EBM-2 cell medium. 25,000 MitoTracker labeled neutrophils were seeded onto the endothelial monolayer (treated with 50ng/ml of TNFα with or without 1μg/ml of LCN2 for 24hr) and incubated for 90 min at 37°C. Media were removed, and each well was washed with media to remove non-adherent neutrophils. The cells were fixed with 4% paraformaldehyde (PFA) for twenty minutes, and examined by fluorescent microscopy. Three images (at 200x magnification) were taken from each well. The number of neutrophils was counted in each field. The assay was performed three times in triplicate wells.

### Endothelial monolayer permeability assay

In vitro endothelial monolayer permeability assay was performed according to previously described methods [22]. HBMECs were seeded on the inner surface of collagen-coated transwell inserts (6.5mm diameter, 0.4μm pore size polycarbonate filter; Corning, Corning, NY), which were placed in wells of a 24-well plate with complete EBM-2 media. When the monolayer of cells was confluent, confirmed by ensuring that it is impermeable to media, the cells were starved for 8h with EBM-2 media without growth supplement and serum before treatment. The cells were treated with 50ng/ml of TNFα with or without 1μg/ml of LCN2 for 24hr before permeability measurement. Permeability was measured by adding 0.1 mg/ml of Fluorescein isothiocyanate (FITC)-labeled dextran (MW, 70,000; Sigma, St. Louis, MO) to the upper chamber, with lower compartment containing fresh serum-free media. After incubation for 20 min, 100μl of sample from the lower compartment was measured for fluorescence at excitation 490nm and emission 520nm. All independent experiments were performed in duplicate or triplicate.

### Western blotting

HBMEC cells were lysed in lysis buffer in the presence of Protease Inhibitor Cocktails (Thermo). Total proteins were harvested after insoluble materials were removed by centrifugation at 14,000 rpm for 20 min at 4°C. Membrane proteins of HBMECs were extracted using ProteoExtract Native membrane Protein Extraction Kit (Millipore). Equal amounts of protein (20 μg/lane) were separated in 4–12% NuPAGE Bis-Tris gels (Invitrogen), and then transferred onto nitrocellulose membranes (Invitrogen). The membranes were blocked with 5% non-fat dry milk and then probed with primary antibodies against VE-cadherin (Abcam), claudin-5 (Abcam), occludin (Abcam), ZO-1 (Abcam), and Na-K-ATPase (Abcam) at 4°C overnight. After washing, the membrane was incubated with horseradish peroxidase-conjugated secondary antibody for 1 hr at room temperature, followed by an enhanced chemiluminescent substrate for detection of HRP (Pierce). The optical density of protein bands was quantified using NIH ImageJ.

### Statistical analysis

All experiments were repeated at least 3 times independently in a randomized and blinded manner. Data were expressed as mean±SD. Quantitative data were analyzed using one-way (SPSS version 16.0). Values of p<0.05 were considered statistically significant.

## Results

### LCN2 levels elevated in the plasma after cerebral ischemia

**Fig 1:**
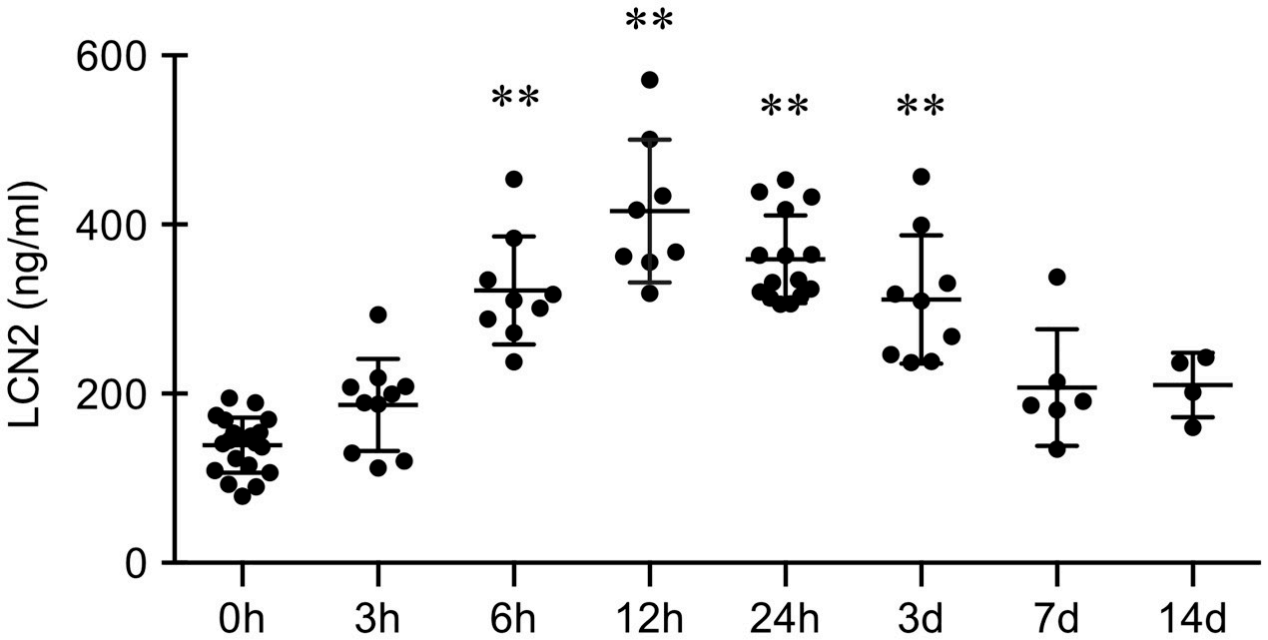
Levels of LCN2 in the plasma before and after cerebral ischemia. Rats were subjected to middle cerebral artery occlusion, and the levels of LCN2 in the plasma were measured using ELISA at different time points. **, p<0.01 compared to 0h.

Rats were subjected to middle cerebral artery occlusion. At different time points after ischemic onset, LCN levels in plasma were measured using ELISA (Fig 1). Plasma levels of LCN2 significantly increased as early as 6 hrs, and peaked at approximately 2-fold over baseline by 12 to 24 hrs after cerebral ischemia. Plasma LCN2 levels remained elevated until 3 days, and then gradually returned to baseline by 7 days. Since LCN2 levels in the blood were significantly elevated in the acute and subacute stage of cerebral ischemia, we asked whether and how LCN2 would affect endothelium, including endothelial proliferation and viability, adhesion, and permeability.

### LCN2 did not affect the proliferation and viability of brain endothelial cells

**Fig 2:**
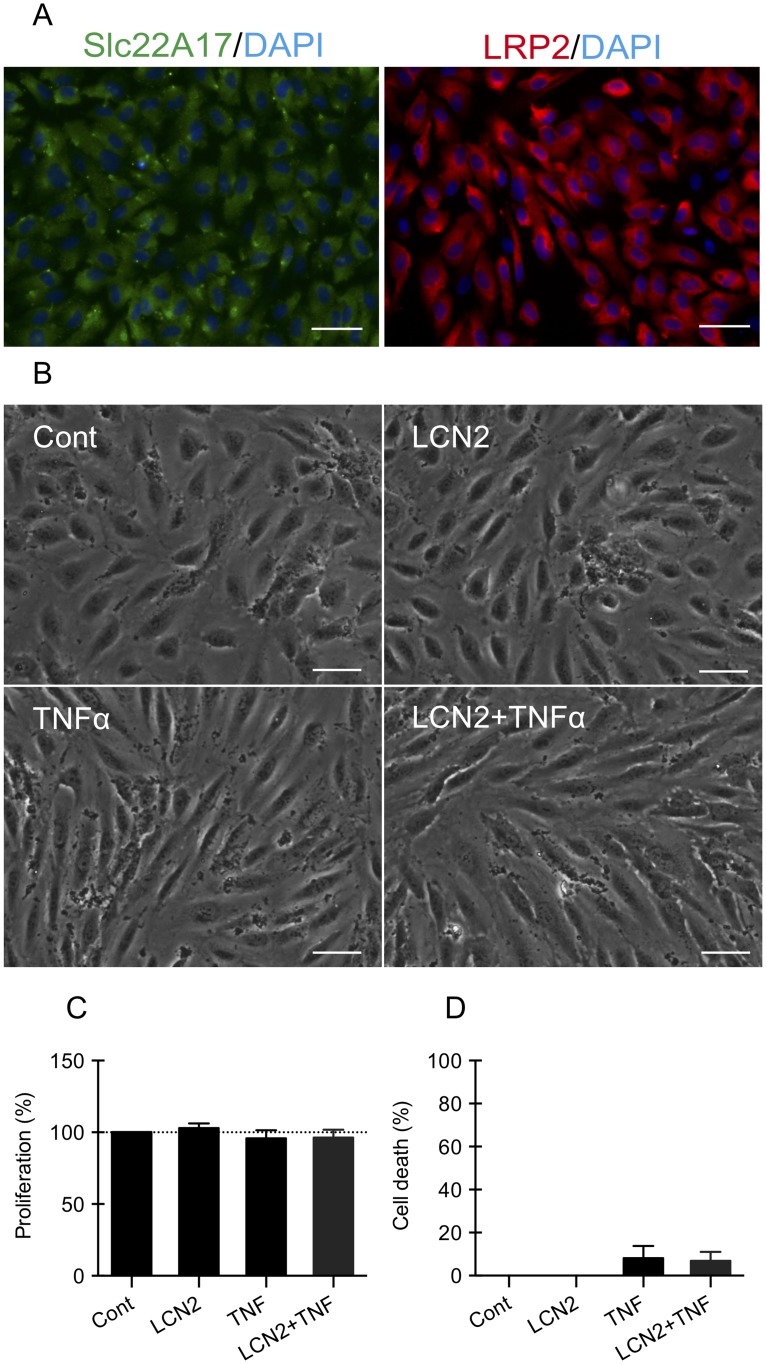
LCN2 did not affect the proliferation and cell viability of the endothelial cells. (A) Immunohistochemistry showed that both of the LCN2 receptors, 24p3R and LRP2, were expressed in human brain endothelial cells. Bar = 50 μm. (B) Morphological change of human brain endothelial cells after TNFα and LCN2 treatment. Bar = 50 μm. (C-D) TNFα treatment w/wo LCN2 did not affect cell proliferation (C, MTT assay) and cell death (D, LDH assay) in human brain endothelial cells. Data were expressed as mean±SD. n = 4 to 5 independent experiments.

First, using immunohistochemistry, we confirmed that both of the LCN2 receptors, 24p3R and LRP2, were expressed in human brain microvascular endothelial cells (Fig 2A). Second, we observed their morphological change after TNFα and LCN2 treatment. Under normal conditions, brain endothelial cells showed a typical “cobblestone” monolayer morphology, and LCN2 treatment alone did not alter the normal cell shape (Fig 2B). After treatment with 50ng/ml of TNFα for 24 hrs, the endothelial cells became spindled with longer cell body, but LCN2 treatment did not restore the spindled endothelial cells back to normal “cobblestone” cell shape (Fig 2B). Third, we investigated whether treatment with TNFα or LCN2 would affect the proliferation and cell viability of the endothelial cells. LCN2 alone or 50ng/ml of TNFα treatment with or without LCN2 did not alter cell proliferation that was measured using MTT assay (Fig 2C). There were also no detectable effects on endothelial cell death and viability measured using the LDH assay (Fig 2D).

### LCN2 had no effect on TNFα-induced increase of neutrophil adhesion

**Fig 3:**
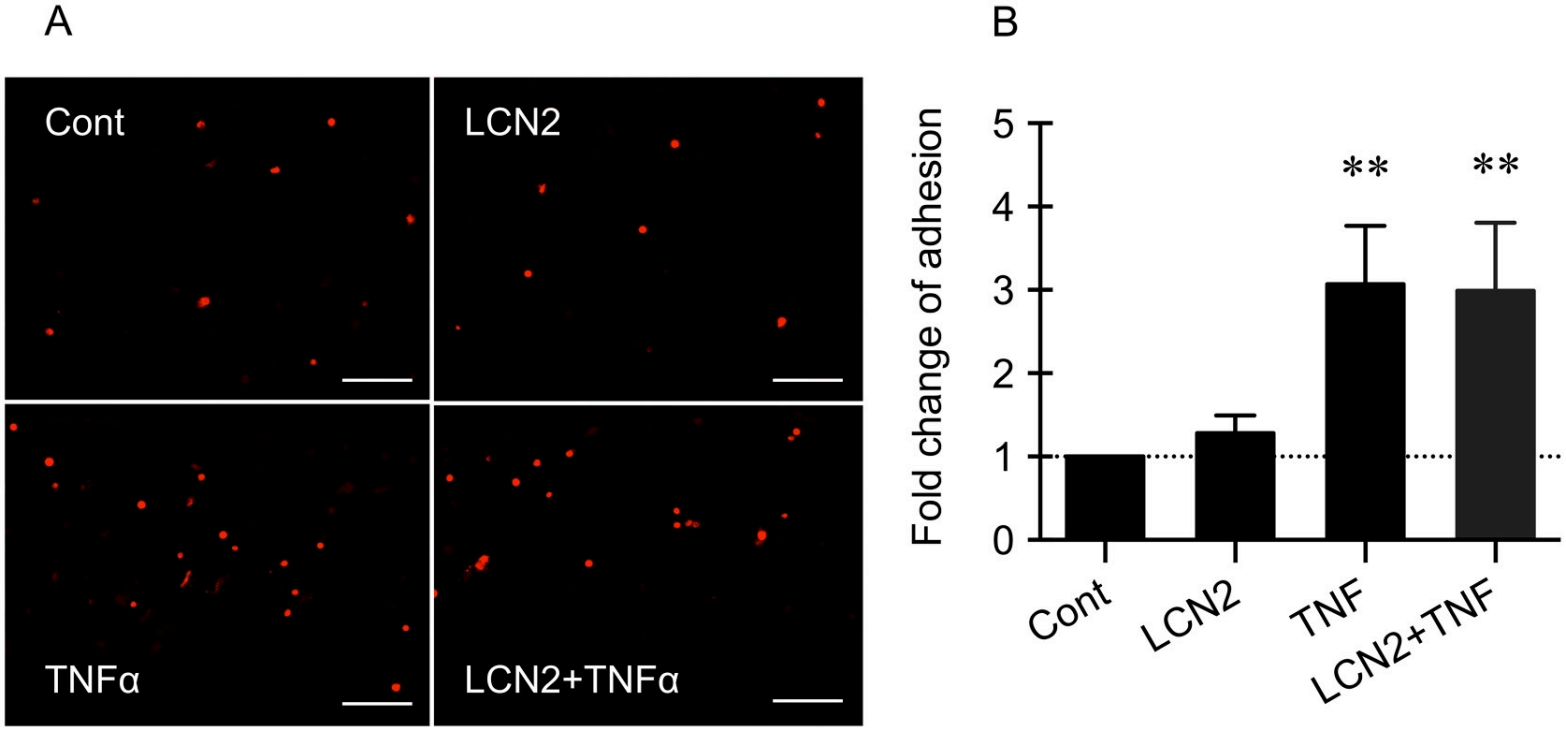
LCN2 had no effect on TNFα-induced increase of neutrophil adhesion. (A) Representative photos of neutrophil adhesion assay. Bar = 50 μm. (B) TNFα treatment significantly increased the number of adherent neutrophils on the endothelial cells. However, LCN2 showed no effect on TNFα induced increase of the number of adherent neutrophils. Data were expressed as mean±SD. **, p<0.01 compared to the control (one-way ANOVA). n = 5 independent experiments.

The adhesion function of endothelial cells was assessed using a standard neutrophil adhesion assay. Representative photos of adherent neutrophils on the endothelial monolayers are shown in Fig 3A. After treatment with TNFα, the number of adherent neutrophils significantly increased by about 3-fold (Fig 3B). However, LCN2 showed no effect on TNFα-induced increase of the number of adherent neutrophils (Fig 3B). LCN2 alone did not affect neutrophil adhesion to the endothelial cells.

### LCN2 rescued VE-cadherin and ZO-1 expression and reduced the increase of endothelial permeability in response to TNFα

**Fig 4:**
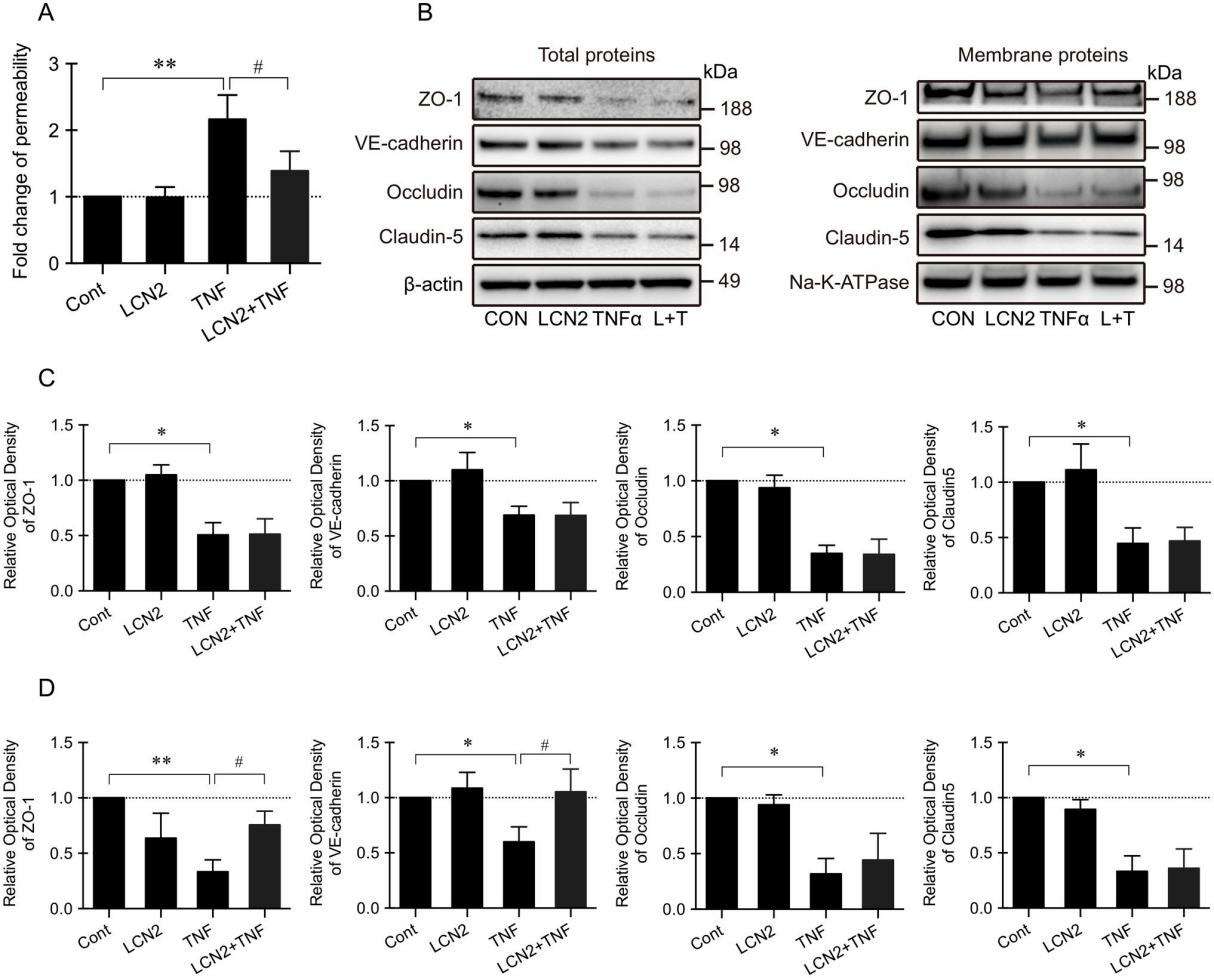
LCN2 reduced the increase of endothelial permeability in response to TNFα. (A) TNFα treatment significantly increased the leakage of 70KD Dextron through the monolayer of endothelial cells, and LCN2 treatment restored TNFα induced increased endothelial permeability. Data were expressed as mean±SD. **, p<0.01 compared to the control; #, p<0.05 compared with TNFα treatment (one-way ANOVA). n = 3 independent experiments. (B) Representative Western blot of total expression and membrane expression of tight junction proteins. (C) TNFα treatment significantly decreased the total protein expression of VE-cadherin, claudin-5, occludin, and ZO-1. *, p<0.05 compared to the control (one-way ANOVA). n = 3 independent experiments. (D) TNFα treatment significantly decreased the expression of VE-cadherin and ZO-1 on the cell membrane, and LCN2 reversed this decrease induced by TNFα treatment. Data were expressed as mean±SD. *, p<0.05, **, p<0.01 compared to the control; #, p<0.05 compared with TNFα treatment (one-way ANOVA). n = 3 independent experiments.

**Fig 5:**
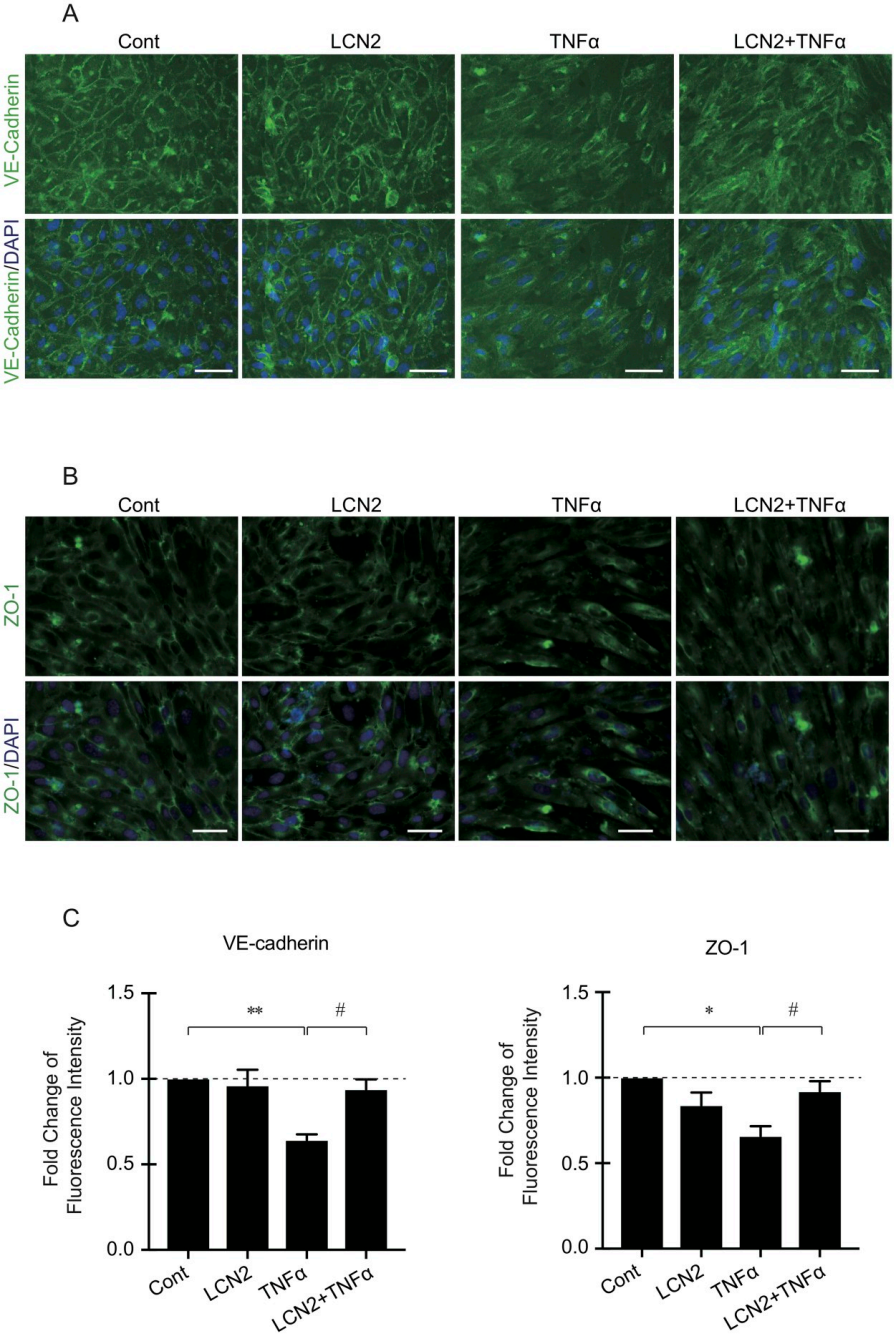
Localization of VE-cadherin and ZO-1 after TNFα or LCN2 treatment using immunocytochemistry. (A-B) Under normal condition, both VE-cadherin (A) and ZO-1 (B) mainly located on the cellular membrane. After TNFα treatment, positive signals of VE-cadherin and ZO-1 immunostaining mainly appeared in the cytoplasm. LCN2 partially restored the distribution of membrane of VE-cadherin and ZO-1. Bar = 50 μm. (C) Bar graph of the fold changes of fluorescence intensity of VE-cadherin and ZO-1. *, p<0.05, **, p<0.01 compared to the control; #, p<0.05 compared with TNFα treatment (one-way ANOVA). n = 3 independent experiments.

A standard transwell assay was used to measure permeability of cultured endothelial cells in vitro. TNFα significantly increased the leakage of 70KD dextran through the endothelial monolayer by about 2-fold (Fig 4A). LCN2 alone did not change endothelial permeability, but LCN2 treatment restored endothelial permeability that was increased by TNFα (Fig 4A). Next, we asked whether responses in endothelial tight junction proteins were consistent with these effects on endothelial permeability (Fig 4B). LCN2 treatment did not change the expression of total and membrane proteins of VE-cadherin, claudin-5, occludin, and ZO-1 (Fig 4C and 4D). However, TNFα treatment significantly decreased the expression of total proteins of VE-cadherin, claudin-5, occludin, and ZO-1 as well as their expression on the cell membrane (Fig 4C and 4D). LCN2 reversed the decreased membrane expression of VE-cadherin and ZO-1 induced by TNFα treatment (Fig 4D). To further confirm the cellular location of VE-cadherin and ZO-1 after TNFα or LCN2 treatment, we performed immunocytochemistry (Fig 5). Under normal conditions, both VE-cadherin and ZO-1 were mainly located on the cell membrane, and LCN2 alone did not change the location of these two proteins. However, after TNFα treatment, there was a reduction in membrane-associated signals of VE-cadherin and ZO-1, and positive signals mainly appeared in the cytoplasm (Fig 5A and 5B). LCN2 seemed partially restore the membrane distribution of membrane of VE-cadherin and ZO-1 that was damaged by TNFα treatment (Fig 5A and 5B). Moreover, TNFα treatment reduced the fluorescence intensity of VE-cadherin and ZO-1, but fluorescence intensity of VE-cadherin and ZO-1 were similar to the control group after co-incubation with LCN2 and TNFα (Fig 5C).

## Discussion

The immune response is a major contributor to the pathophysiology of ischemic stroke. Under normal conditions, the brain is a relatively “immune-privileged” organ, which reflects both the tightness of the BBB as well as the low baseline expression of adhesion molecules and receptors involved in leukocyte diapedesis [23, 24]. After stroke, endothelial adhesion and permeability are significantly perturbed, and these changes facilitate and amplify neuroinflammation. Our present study suggests that LCN2 might represent an endogenous “help-me” signal that attempts to defend against endothelial injury. LCN2 is markedly increased in blood during subacute and acute phases after focal cerebral ischemia in rats, and LCN2 is able to ameliorate inflammatory disruptions in tight junction proteins and BBB permeability in vitro.

LCN2 is an acute-phase protein demonstrating increased systemic levels in many human diseases, especially inflammation, infection, and ischemia [25]. Although originally identified in neutrophil granules [26], lipocalin-2 is expressed in many tissues, including bone marrow, liver, spleen, heart, lungs, thymus, and kidney [27]. Its expression can be induced in many organs during inflammation and after injury. Lipocalin-2 plays a key role in the innate immune to limit bacterial growth by binding to iron-laden bacterial siderophores [28]. Lipocalin-2 is a promising biomarker of acute kidney injury and chronic kidney diseases [29]. In a mouse model of renal damage, LCN2 protects the kidney against ischemia-reperfusion [30]. In gastrointestinal injury, lipocalin-2 facilitates mucosal regeneration by promoting cell migration [31]. LCN2 may function as a proliferation and differentiation factor. Macrophages that overexpress anti-inflammatory interleukin-10 (IL-10) are protective in rat models of kidney dysfunction via iron-mediated upregulation of LCN2 and its receptors, eliciting both anti-inflammatory and proliferative responses [32]. In the CNS, LCN2 is associated with Alzheimer’s disease pathology in human postmortem brain [33]. In rat brain, LCN2 mRNA and protein is upregulated after neuronal injury induced by kainite [34] or after neuroinflammation induced by systemic lipopolysaccharide injections [35]. In human stroke, serum levels of LCN2 progressively increased following acute ischemia and transient ischemic attacks [36, 37].

Following cerebral ischemia, neutrophils are the first blood-borne cells to respond the injury and arrive at the ischemic area. Neutrophils infiltration in brain parenchyma reached peak at approximately 24–72 hrs after ischemia onset and then decrease thereafter but immune mobilization will continue for days to weeks afterwards [38–41]. In the four subsequent phases of the immigration of attracted neutrophils, i.e. rolling adhesion, tight adhesion and diapedesis/migration, and finally crossing the BBB, neutrophil adhesion is an important step [42, 43]. Neutrophils attach to the endothelium via the interaction of various adhesion molecules including the selectins, intracellular cell adhesion molecule-1 (ICAM-1) and integrins. In our studies, after 24 h incubating cultured endothelial cells with TNFα, the number of neutrophils that attached to the monolayer of endothelial cells dramatically increased. This effect might be due to upregulated expression of adhesion receptors in endothelial cells by TNFα, including E-selectin, and ICAM-1 and VCAM-1, and/or changed localization of some other adhesion molecules in endothelial cells by TNFα, such as P-selectin [44]. However, LCN2 treatment did not affect the attachment of neutrophils to the endothelial cells both under normal condition and after TNFα treatment. Neutrophils have controversial effects in stroke. Some studies correlated neutrophils to ischemia-induced brain injury [45, 46]. In animal models of cerebral ischemia, reducing neutrophil infiltration by blocking adhesion molecules reduced brain edema, neurological deficits and mortality [47–49]. However other studies showed that neutrophil infiltration mitigated stroke injury [50, 51]. Recent evidence suggested that neutrophils might adopt a pro-inflammatory N1 phenotype or an anti-inflammatory N2 phenotype in the CNS [52]. In a mouse model of permanent focal cerebral ischemia, N2 polarization of neutrophil was a crucial event for resolution of inflammation and neuroprotection [53].

Peripheral immune cell infiltration is also facilitated by the BBB dysfunction after ischemic stroke [54]. BBB plays a vital role in separating the CNS from the blood and immune system, regulating the trafficking of fluid, solutes and cells at the blood-brain interface, and maintaining the homeostasis of the CNS [24, 55]. BBB dysfunction, i.e. loss of structural integrity and normal functions, is a prominent pathological characteristic of many neurological disorders, including stroke [56]. After ischemic stroke, intravascular proteins, fluid, and immune cells extravasate into brain parenchyma across impaired BBB, resulting in brain edema and expansion of tissue damage [57, 58]. The BBB is comprised of endothelial cells, the basement membrane, pericytes, and the end-feet of astrocytes [55]. Although pericytes and astrocytes play a major regulatory role, the cerebral endothelial cells are the foundation of the BBB and intact endothelium plays a critical role in the barrier function of BBB. The junctional complexes between ECs include tight junctions (TJs) and adherens junctions (AJs). TJs constitute the primary structure of the paracellular barrier between ECs and these proteins, including occluding, claudins, junctional adhesion molecules, and zonula occludens (ZO) proteins, are central regulators of BBB permeability [55, 59]. AJs are located below the TJs, and the main components of AJs are cadherin, VE cadherin and associated scaffolding proteins catenin [60]. AJs stabilize endothelial cell-cell interactions and also help control paracellular permeability [60]. In our present study, TNFα treatment significantly increased the paracellular permeability of endothelial cells by destroying both tight junctions and adherens junctions between endothelial cells. LCN2 could at least partially restore the normal structure of the junctional complex of endothelial cells, and reduced the increase of paracellular permeability induced by TNFα.

Taken together, our findings suggested that the acute elevations of LCN2 levels in blood shortly after the onset of acute ischemic stroke may represent an endogenous help-me response that can help to reduce damage within the junctional complex of endothelial cells and rescue BBB permeability. However, there are several caveats in this proof-of-principle study. First, the infiltration of peripheral immune cells into brain parenchyma after ischemia is a complex process with multifactorial mechanisms. Here, we observed that LCN2 rescued endothelial permeability but not adhesion. But beyond adhesion and permeability, the effects of LCN2 on other mechanisms involved leukocyte transmigration should be explored. Second, trans-endothelial transport not only occurs paracellularly but also via trans-cellular routes. Recent evidence suggests that fluid, solutes, and even circulating cells can cross the endothelium by passing through an individual endothelial cell body. How LCN2 affects the trans-cellular mechanisms of leukocyte passage should be considered carefully. Third, other components of the neurovascular unit, such as pericytes and astrocytes, also play important roles in maintaining normal BBB functions [61, 62]. The effects of LCN2 on the functions of the pericytes or astrocytes should also be rigorously dissected. Finally, although LCN2 ameliorated the inflammatory disruption in tight junction proteins and permeability, it did not appear to alter endothelial adhesion. Further in vivo studies are warranted to assess how LCN2 affects neuroinflammation in stroke.

In summary, our proof-of-principle study is consistent with the idea that LCN2 may act as an endogenous help-me signal in the injured CNS. A deeper dissection of LCN2 mechanisms in endothelium may lead to new directions and targets to rescue BBB integrity and dampen neuroinflammation after stroke.
